# Thermodynamical framework for effective mitigation of high aerosol loading in the Indo-Gangetic Plain during winter

**DOI:** 10.1038/s41598-023-40657-w

**Published:** 2023-08-22

**Authors:** Prodip Acharja, Sachin D. Ghude, Baerbel Sinha, Mary Barth, Gaurav Govardhan, Rachana Kulkarni, Vinayak Sinha, Rajesh Kumar, Kaushar Ali, Ismail Gultepe, Jean-Eudes Petit, Madhavan Nair Rajeevan

**Affiliations:** 1 Indian Institute of Tropical Meteorology, Ministry of Earth Sciences, Pune, India; 2 Laboratoire des Sciences du Climat et de l’Environnement, LSCE, CNRS, Gif-sur-Yvette, France; 3 Department of Earth and Environmental Sciences, Indian Institute of Science Education and Research Mohali, Sahibzada Ajit Singh Nagar, Punjab India; 4 National Center for Atmospheric Research, Boulder, CO 80307 USA; 5 Savitribai Phule Pune University, Pune, 411007 India; 6 Engineering and Applied Science, Ontario Technical University, Oshawa, ON Canada; 7 Civil and Environment Eng and Earth Sciences, University of Notre Dame, Notre Dame, IN USA; 8 Ministry of Earth Science, Lodhi Road, New Delhi, India

**Keywords:** Environmental chemistry, Atmospheric chemistry

## Abstract

The Indo-Gangetic Plain (IGP) experiences severe air pollution every winter, with ammonium chloride and ammonium nitrate as the major inorganic fractions of fine aerosols. Many past attempts to tackle air pollution in the IGP were inadequate, as they targeted a subset of the primary pollutants in an environment where the majority of the particulate matter burden is secondary in nature. Here, we provide new mechanistic insight into aerosol mitigation by integrating the ISORROPIA-II thermodynamical model with high-resolution simultaneous measurements of precursor gases and aerosols. A mathematical framework is explored to investigate the complex interaction between hydrochloric acid (HCl), nitrogen oxides (NO_x_), ammonia (NH_3_), and aerosol liquid water content (ALWC). Aerosol acidity (pH) and ALWC emerge as governing factors that modulate the gas-to-particle phase partitioning and mass loading of fine aerosols. Six "sensitivity regimes" were defined, where PM_1_ and PM_2.5_ fall in the "HCl and HNO_3_ sensitive regime", emphasizing that HCl and HNO_3_ reductions would be the most effective pathway for aerosol mitigation in the IGP, which is ammonia-rich during winter. This study provides evidence that precursor abatement for aerosol mitigation should not be based on their descending mass concentrations but instead on their sensitivity to high aerosol loading.

## Introduction

High aerosol loading is a significant cause of millions of premature deaths around the world, and mitigating air pollution is a major concern for researchers over the globe^1,2^. However, to regulate or mitigate high aerosol loading, it needs to be measured, monitored, and investigated thoroughly. Despite extensive efforts, the understanding of the physical, chemical, and thermodynamical properties of atmospheric constituents has not yet reached a point where aerosol mitigation can be done precisely and optimally^3–6^. There is a growing need to better understand the aerosol properties as they severely affect the ecosystem, human health, and the environment.

The Indo-Gangetic Plain (IGP) is one of the most polluted regions in the world^7–10^. The mass loading of PM_1_ and PM_2.5_ often exceeds 400–600 µg m^−3^ for the short-term (few hours), particularly during traffic rush hours and nighttime during winter^11,12^. Studies have shown that the organic fraction is generally responsible for more than 50% of PM_1_ mass globally, and the inorganic fraction of fine aerosol is composed of sulfate, nitrate, and ammonium (SNA)^13–16^. But in contrast, few studies have also shown that more than half of total aerosol loading is inorganic in nature during peak pollution episodes (Table S1), pointing out the necessity of investigating the role of physicochemical and thermodynamical properties of atmospheric constituents in contrasting atmospheric conditions. For instance, Gani et al.^17^ have shown that during peak pollution periods of January 2018, the inorganic fractions contributed nearly 60% of the total PM_1_ mass loading in Delhi. These inorganic aerosols are primarily composed of chloride, sulfate, nitrate, and ammonium (CSNA)^18,19^.

The observed hourly chloride concentrations many times exceed 100 µg m^−3^, considered to be among the highest reported anywhere in the world^20^. The gas-phase ammonia (NH_3_) is also found to be very high, which significantly affects the secondary aerosol formation in winter. Several recent studies have investigated the sensitivity of aerosols to the reduction in precursor gases in China, the USA, and Europe^4^, but studies conducted in India have not yet been performed. Extensive efforts are needed to improve our scientific understanding of the effective regulation of aerosol loading in the IGP.

In this study, we aim to provide new mechanistic insights into aerosol formation and mitigation by integrating the ISORROPIA-II thermodynamic equilibrium model with the dataset of precursor gases (HCl, HNO_3_, and NH_3_) and inorganic constituents (Cl^−^, NO_3_^−^, SO_4_^2−^, Na^+^, NH_4_^+^, K^+^, Ca^2+^, and Mg^2+^) of PM_1_ and PM_2.5_ acquired using the first deployment of the MARGA-2S instrument in the IGP (Supplementary text S1). We explore a mathematical framework to investigate the sensitivity of gas-to-particle partitioning of aerosols to different parameters like gaseous precursor concentrations (HCl, HNO_3_, and NH_3_), pH, and ALWC, using sigmoidal curves and "sensitivity regimes" of aerosols. To our knowledge, this is the first attempt to investigate the thermodynamic control of aerosols over the Indian region, and the "thermodynamical roadmap" advanced in this study could provide effective and targeted mitigation strategies in the IGP.

## Results

### Evaluation of the ISORROPIA-II model simulations and sensitivity analysis

The ISORROPIA-II model was run in the "forward" mode using the hourly measured total (gas + particle) species concentrations instead of only particle-phase concentrations as input, as it is considered to be more accurate^21,22^. As shown in Figure S1, the predicted PM_1_ NH_4_^+^ (r = 0.97), NO_3_^−^ (r = 0.93), Cl^−^ (r = 0.98), PM_2.5_ NH_4_^+^ (r = 0.87), NO_3_^−^ (r = 0.98), Cl^−^ (r = 0.99), and gas-phase NH_3_ (r = 0.93) showed very good correlation with the measured concentrations, confirming the reliability of the ISORROPIA-II model simulations at a range of temperatures (278–298 K).

The data points are color-coded by ambient temperatures to investigate how temperature variability can alter the ISORROPIA-II predicted gas and particle-phase concentrations. The pH and ALWC of PM_1_ and PM_2.5_ were estimated during 19 December 2017 to 10 February 2018. The predicted pH of PM_1_ varied between 2.2 to 5.6, and the mean PM_1_ pH (average ± SD) was 4.5 ± 0.5. The PM_2.5_ pH ranged from 2.5 to 6.5, with a mean value of 4.6 ± 0.5. The predicted pH of PM_2.5_ was similar to the measured PM_2.5_ pH of 4.6 ± 0.5 over Delhi, estimated during the Winter Fog EXperiment (WiFEX) campaign period of 2015–16, underscoring the reliability and accuracy of the ISORROPIA-II model simulation^20,23^.

In spite of the high reliability of the ISORROPIA-II model simulations (Figure S1), it is essential to investigate the uncertainties in observations while using them as inputs to model simulations. There could be many sources of errors in the measurements, like gas-aerosol collection efficiency, internal standard variability, and reproducibility of the chromatogram peak integration in the MARGA-2S. These errors sometimes could propagate to cause significant uncertainties in the model simulations.

It can be seen that, at almost all times during the study, the concentrations of the measured parameters were much higher than the range of errors, causing them to be unlikely the primary source of bias in the analysis. It is crucial to note that the analytic error in monitoring individual species cannot be evaluated in the absence of specifically designed quality-control experiments. However, to ensure the quality of the data, observational hours were excluded from the study when measurements did not meet the quality assurance and quality check (QA/QC) criteria^19^. As a result, only 1100 h of correct observational datasets were used for the model simulations, and other datasets are not included in the analysis of this paper.

The major errors inherent with thermodynamic equilibrium modeling are derived from two assumptions, i.e., internal mixing and gas-aerosol equilibria. Among different factors that contribute to the uncertainty in the model simulations, input parameters like gas concentrations, aerosol composition, temperature, and relative humidity are considered to be the most crucial. Uncertainties in these input parameters could cause biases in predicted parameters like aerosol acidity (pH), aerosol liquid water content (ALWC), and gas-aerosol phase partitioning (ε).

To quantitively investigate this, we carried out simulations with different sets of gas and particle-phase inputs. The analysis has also considered the limit of detection of all gas and aerosol species by the MARGA instrument (Table S3). The statistical variability was evaluated by checking the mean bias (MB), normalized mean bias (NMB), and root mean square error (RMSE) following Eqs. (S7–S9). As many previous studies in India considered fixed concentrations of HCl + Cl^−^ (25 µg m^−3^) as model input due to a lack of gas-phase HCl observations in their studies^24,25^, we also fixed the input concentrations to 25 µg m^−3^. To make the analysis more robust, we fixed the concentrations of all three gas + aerosol constituents, i.e., HCl + Cl^−^, HNO_3_ + NO_3_^−^, and NH_3_ + NH_4_^+^. The parameters in Table S4 illustrate the uncertainty in aerosol acidity (pH) and aerosol liquid water content (ALWC) due to these fixed input concentrations.

As ALWC governs the presence of ions and the water uptake of different hygroscopic inorganic constituents, it is crucial to investigate the uncertainty in ALWC due to different assumptions in model input, especially in a region like Delhi, where ALWC is very high during winter, and the heterogeneous and multiple-phase reactions govern during peak-pollution events. The fixed concentration of HCl + Cl^−^ = 25 µg m^−3^ resulted in NMB of − 8.34% and − 26.61% for PM_1_ and PM_2.5_ ALWC, respectively. The RMSE is 149.69 µg m^−3^ and 264.98 µg m^−3^ for fixed total chloride input concentrations. The RMSE in ALWC due to fixed total nitrate (HNO_3_ + NO_3_^−^ = 25 µg m^−3^) and total ammonium (NH_3_ + NH_4_^+^  = 25 µg m^−3^) concentrations are 46.8 µg m^−3^, 62.49 µg m^−3^ and 93.70 µg m^−3^, 307.81 µg m^−3^ for PM_1_ and PM_2.5_ respectively. These high uncertainties could significantly alter the predicted gas-aerosol phase partitioning and particle-phase loading in the atmosphere.

The aerosol acidity (pH) of PM_1_ and PM_2.5_ also shows significant bias due to assumed fixed input concentrations (Table S4). The RMSE of PM_1_ and PM_2.5_ pH due to fixed total chloride input concentration is 0.42 and 0.55 respectively, which can significantly alter the phase-partitioning and particle-phase chloride loading.

Furthermore, we have extended our efforts by conducting an uncertainty analysis in the model simulations to account for the inherent measurement uncertainties associated with the MARGA-2S trace gas and aerosol species measurements. This undertaking involves the consideration of three distinct input values, encompassing the average and 95% confidence interval upper and lower limits, for all species under investigation in the "forward" mode model simulations.

Of notable importance are the primary input parameters (95% confidence intervals indicated in parentheses): TCl = 21.78 (20.59–22.98) µg m^−3^, TNO_3_ = 19.74 (19.17–20.31) µg m^−3^, TNH_4_ = 43.43 (42.28–45.58) µg m^−3^, SO_4_^2−^ = 11.36 (10.99–11.73) µg m^−3^ for PM_1_, and TCl = 39.71 (37.65–41.77) µg m^−3^, TNO_3_ = 31.65 (30.80–32.50) µg m^−3^, TNH_4_ = 58.02 (56.62–59.42) µg m^−3^, and SO_4_^2−^ = 20.34 (19.52–21.15) µg m^−3^ for PM_2.5_. We have included the uncertainties associated with the model-derived aerosol liquid water content (ALWC) and aerosol acidity (pH) values for both PM_1_ and PM_2.5_ in Table S4. These results collectively emphasize the robustness and reliability of the ISORROPIA-II model simulations.

In regions like Europe, where HCl and Cl^−^ concentrations are very low, the assumption of fixed HCl + Cl^−^ may not have a grave impact, but in a region like Delhi, where observed HCl + Cl^−^ concentrations varied between 2.11–112.21 µg m^−3^ and 2.31–186.31 µg m^−3^ for PM_1_ and PM_2.5_ respectively, the consideration of actual ambient concentrations in ISORROPIA-II model simulations seems to be of utmost importance. The fixed concentration of 25 µg m^−3^ cannot accurately represent real-world conditions. Previous studies have also emphasized that when gas-phase data are not available, running ISORROPIA-II in the forward mode, with only aerosol concentrations as input, results in the repartitioning of ammonia in the model, causing bias in the predicted pH and ALWC^3,21^. These sensitivity analyses with fixed input concentrations of total chloride, nitrate, and ammonium illustrate the necessity of accounting for the actual gas and aerosol species concentrations instead of fixed input concentrations, especially in the IGP during winter.

### Sensitivity of gas-to-particle phase partitioning (ε) to pH and ALWC during winter

As the high ammonium chloride concentration is a special characteristic of air pollution in India, and particle-phase chloride and nitrates are the major contributors to the inorganic fraction of the particulate matter burden in Delhi, it is crucial to explore their variability in prevailing meteorological conditions^17,19,24^. But under wintertime ambient meteorological conditions, pure hydrochloric acid, nitric acid, or water particles are difficult to form due to the higher vapor pressure of HCl and HNO_3_. Instead, ammonium nitrate (NH_4_NO_3_) and ammonium chloride (NH_4_Cl) form with significantly lower vapor pressure and has longer atmospheric residence time compared to the corresponding precursor gases like HNO_3_, HCl, and NH_3_^26^. The average chloride partitioning ratio ε(Cl^−^) of PM_1_ and PM_2.5_ was 0.93 ± 0.09 and 0.96 ± 0.07 respectively (Table S2), implying the dominant presence of chloride in the particle phase during winter. The ε(Cl^−^) was 0.4 at RH ≤ 50%, which sharply increased to 0.95 at RH ≥ 80%, showing the enhanced phase-partitioning to the particulate chloride phase. The increased phase-partitioning of total chloride (HCl + Cl^−^) in highly humid conditions can further promote chloride formation caused by increased ALWC. The enhanced ALWC increases pH by dilution, further increasing total chloride partitioning and significantly increasing PM_1_ and PM_2.5_ chloride in a positive feedback loop^22,27^.

It has been shown that aerosols with higher chloride mass fraction uptake more water than those with less chloride mass fraction under prevailing RH conditions^24,28^. This is due to the co-condensation of HCl, NH_3_, and water, as the gas-phase HCl gets dissolved in aerosol water, dissociates, and then equilibrates with ammonia to form ammonium chloride, stabilizing chloride in the particle phase^26,29^. This particle-phase chloride can absorb even more water from the air, leading to enhanced growth of aerosols into fog droplets during winter and augmenting the particle mass loading^24^. These results demonstrate the role of ALWC in the phase-partitioning of PM_1_ and PM_2.5_ aerosols, which needs to be thoroughly investigated for a deeper understanding of the complex thermodynamical control of high aerosol loading.

The average ε(NO_3_^−^) of PM_1_ and PM_2.5_ was 0.83 ± 0.11 and 0.89 ± 0.08 respectively, showing the dominance of particle-phase nitrate over gas-phase HNO_3_ during winter. The high particle-phase nitrate concentrations significantly impact the total aerosol loading as the presence of more ammonium nitrate reduces the deliquescence relative humidity (DRH) for single-component aerosols and mutual deliquescence relative humidity (MDRH) for multicomponent aerosols resulting in the formation of more secondary aerosols in polluted conditions^30–32^. DRH and MDRH are the critical values of relative humidity, above which the water uptake of single component and multicomponent aerosols drastically increases, leading to increased surface area for heterogeneous reactions, enhanced aqueous phase reaction rates, and uptake coefficients of trace gases^31,33^. This, in turn, governs the atmospheric lifetime of both gas and aerosol phases.

To comprehend the importance of DRH or MDRH, we can see that sea salt particles contain highly hygroscopic salt magnesium chloride (MgCl_2_) having DRH of ~ 32%, due to which sea salt particles deliquesce at very low RH. This makes the marine atmosphere hazier than continental air at the same RH and ambient temperature (T). So, the presence of salts with less DRH or MDRH will make the atmosphere hazier than those with less hygroscopic salts like ammonium chloride and ammonium nitrate (38). As the efflorescence RH (ERH) of these aerosols are generally low and rarely reached by the ambient RH during winter, these species primarily remain in the aqueous or metastable phase^34^.

It is to be noted that, the solubility of ammonia is fairly weak, highly temperature-dependent, and greatly influenced by the aerosol pH and effective Henry's law constant. The average ε(NH_4_^+^) for PM_1_ and PM_2.5_ was 0.42 ± 0.17 and 0.55 ± 0.15 respectively, attributed to the substantial excess ammonia left in the gas-phase. Due to this, several studies have highlighted that pollution control strategies in India should be directed at reducing high NH_3_ concentrations^25,35^. Here, in this study, we did not investigate the effect of pH and ALWC on the phase partitioning of sulfate ε(SO_4_^2−^). This is because the sulfate was considered to be majorly present in the ammonium sulfate form, which has a very low volatility in the wintertime ambient temperature range (38). So, this study focuses on investigating the phase partitioning of semi-volatile species like ammonium chloride and ammonium nitrate during winter.

Due to the significantly different particulate matter (PM) burden, PM chemical composition, particle number size distribution, and meteorological conditions between Delhi and other parts of the globe, these phase-partitioning ratios in Delhi are in significant contrast^24,28^. For instance, ε(NO_3_^−^) was reported to be 0.26 ± 0.15 and 0.39 ± 0.16 in the USA, and ε(NH_4_^+^) was 0.2 ± 0.1 in China, but in India, these ratios are significantly different (Table S2)^36,37^. The extremely high ALWC in ammonia-rich Delhi triggers the water uptake, causing an enhanced gradient of water activity and water affinity between aerosols and their surroundings, which acts as the major driving force for enhanced secondary inorganic aerosol (SIA) formation during winter.

We estimated the sensitivity of the phase partitioning of nitrate [ε(NO_3_^−^)], chloride [ε(Cl^−^)], and ammonium [ε(NH_4_^+^)] to pH, ALWC, and T^37–39^ following the methodology given in Guo et al.^37^ as:

1 $$\varepsilon \left({NO}_{3}^{-}\right)=\frac{{H}_{{HNO}_{3}}^{*} R T {ALWC}_{i}\times 0.987 \times {10}^{-14}}{{\gamma }_{{H}^{+}}{\gamma }_{{NO}_{3}^{-}}{10}^{-pH}+ {H}_{{HNO}_{3}}^{*} R T {ALWC}_{i}\times 0.987 \times {10}^{-14}}$$

2 $$\varepsilon \left({Cl}^{-}\right)=\frac{{H}_{HCl}^{*} R T {ALWC}_{i}\times 0.987 \times {10}^{-14}}{{\gamma }_{{H}^{+}}{\gamma }_{{Cl}^{-}}{10}^{-pH}+ {H}_{HCl}^{*} R T {ALWC}_{i}\times 0.987 \times {10}^{-14}}$$

and,

3 $$\varepsilon \left({NH}_{4}^{+}\right)=\frac{\frac{{\gamma }_{{H}^{+}} {10}^{-pH}}{{\gamma }_{{NH}_{4}^{+}}} {H}_{{NH}_{3}}^{*} R T {ALWC}_{i}\times 0.987 \times {10}^{-14}}{1+\frac{{\gamma }_{{H}^{+}} {10}^{-pH}}{{\gamma }_{{NH}_{4}^{+}}} {H}_{{NH}_{3}}^{*} R T {ALWC}_{i}\times 0.987 \times {10}^{-14}},$$

where γ is the activity coefficient of protonated species in the aqueous medium, and ALWC_i_ is the water associated with the inorganic constituents (µg m^−3^). H^*^ is the equilibrium constant of HNO_3_, HCl, and NH_3_ adopted from ^40,41^ using molality-based units of mol^2^ kg^−2^ atm^−1^^42,43^. R is the universal gas constant (8.314 J K^−1^ mol^−1^), and the value 0.987 is for the transformation of 1 atm to 1 bar. The equations describe the HNO_3_–NO_3_^−^, NH_3_–NH_4_^+^, and HCl–Cl^−^ partitioning, and the estimated values of ALWC_i_ and T were used to evaluate the phase-partitioning of ε(NO_3_^−^), ε(Cl^−^), and ε(NH_4_^+^) at different pH regimes.

**Figure 1:**
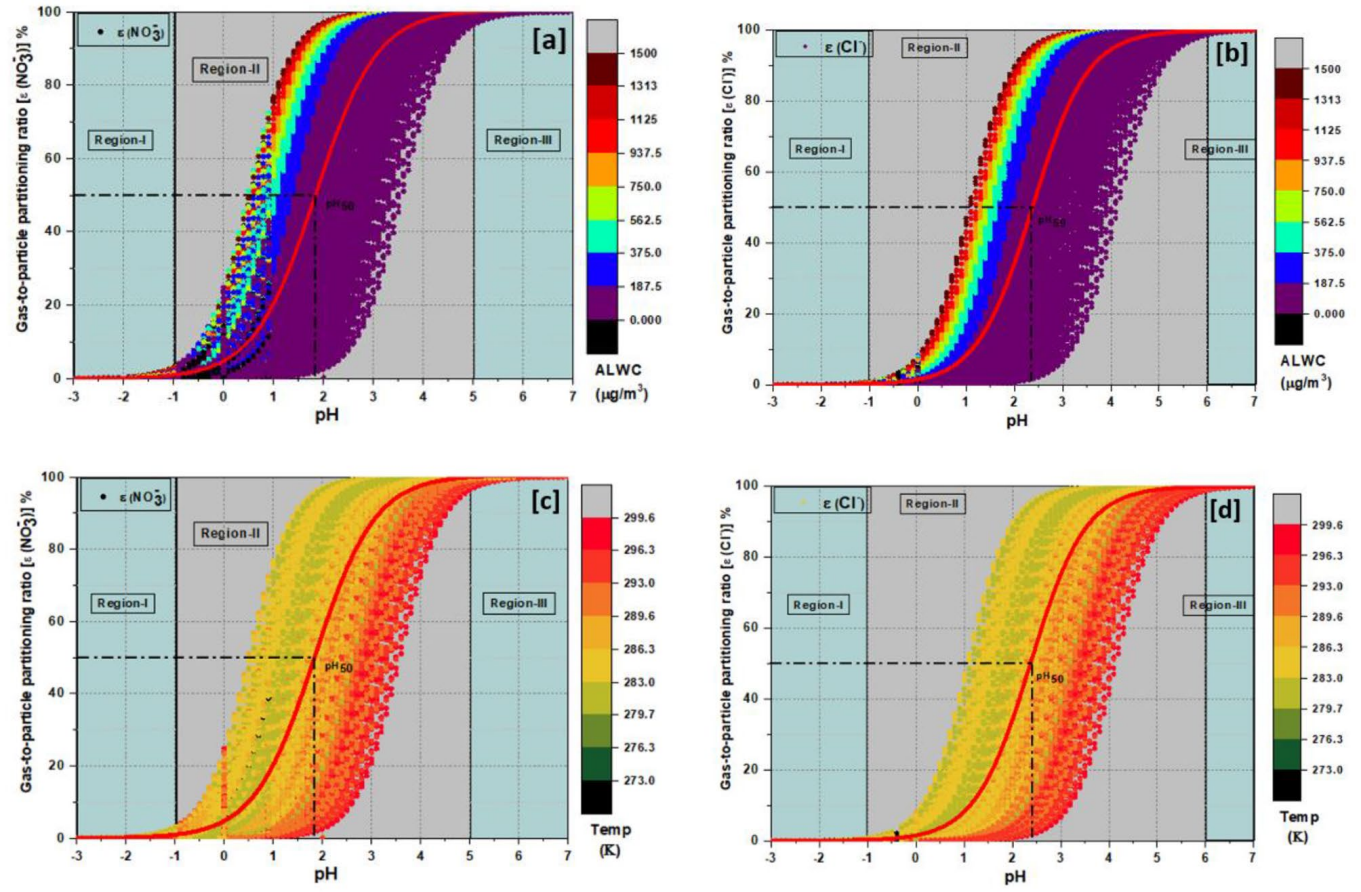
The gas-to-particle partitioning ratio of (**a**,**c**) ε(NO_3_^−^), (**b**,**d**) ε(Cl^−^) with pH are shown by the sigmoidal (S) curves. Three zones are defined, where ε(NO_3_^−^) and ε(Cl^−^) vary between the complete gas-phase (ε ~ 0; blue-color zone, region I) and complete particle-phase (ε ~ 100%; blue-color zone, region III). A characteristic pH (pH_50_) has been defined where total nitrate and chloride remained 50% in the gas-phase and 50% in the particle-phase (gray-color zone, region II). The 1100 hourly data points are color coded with ALWC (1a, 2b) and temperature (1c, 2d) to investigate the impact of ALWC and temperature on the phase-partitioning ratio (ε). The ALWC color bar shows how positively ALWC impacts the phase-partitioning, whereas the temperature color bar shows its inverse relationship with the particle-phase loading over a location.

Figure 1a–d show the variability of gas-to-particle partitioning of nitrate and chloride with pH, ALWC, and T following Eqs. (1–3). Three prominent zones are shown, in which ε(Cl^−^) and ε(NO_3_^−^) vary between the complete gas-phase (ε ~ 0%) to the complete particle-phase (ε ~ 100%). In  region I, ε(Cl^−^) and ε(NO_3_^−^) asymptotically approach 0, and the total species primarily remain in the gas-phase. In  region III, ε asymptotically approaches 1, and the whole species is in the particle phase, whereas in  region II, ε varies between 0 and 1, and the species remain as a mixture of gas and particle-phase. A thermodynamical sweet spot, pH_50_ is defined, where ε(Cl^−^) and ε(NO_3_^−^) are 0.5, and total chloride and nitrate remain 50% in the gas-phase and 50% in the particle phase.

The 1100 hourly data points are color coded with ALWC (1a, 1b) and temperature (1c, 1d) to investigate the impact of ALWC and temperature on the phase-partitioning ratio (ε). The ALWC color bar shows how positively ALWC impacts the phase-partitioning, whereas the temperature color bar shows its inverse relationship with the particle-phase loading over a location. The ALWC color-coded data points show the variability in ε due to variation in ALWC at a constant pH, indicating the significance of ALWC in modulating the particle-phase loading. From Fig. 1a, it can be seen that ε(NO_3_^−^) can vary between 70 and 100% at pH ≈ 4, due to variability in ALWC.

The red line is the sigmoidal curve (S) fitting, which depicts the sensitivity of ε to pH and ALWC over a location. The estimated average pH of PM_1_ and PM_2.5_ of 4.49 and 4.58, respectively (“Formulation of "sensitivity regimes" of aerosols in the IGP”), falls on the flat side of the S curves in the blue-color zone. Here, chloride and nitrate remain almost exclusively in the particle-phase, and ammonia remains primarily in the gas-phase. The possibility that emerges as the pathway to modulate high aerosol concentrations is reducing precursors like HCl and HNO_3_, which is feasible as it would not adversely affect agricultural productivity and potentially impact the ecosystem.

### Aerosol mass loading sensitivity to HCl, HNO_3_, and NH_3_ perturbations in the IGP

**Figure 2:**
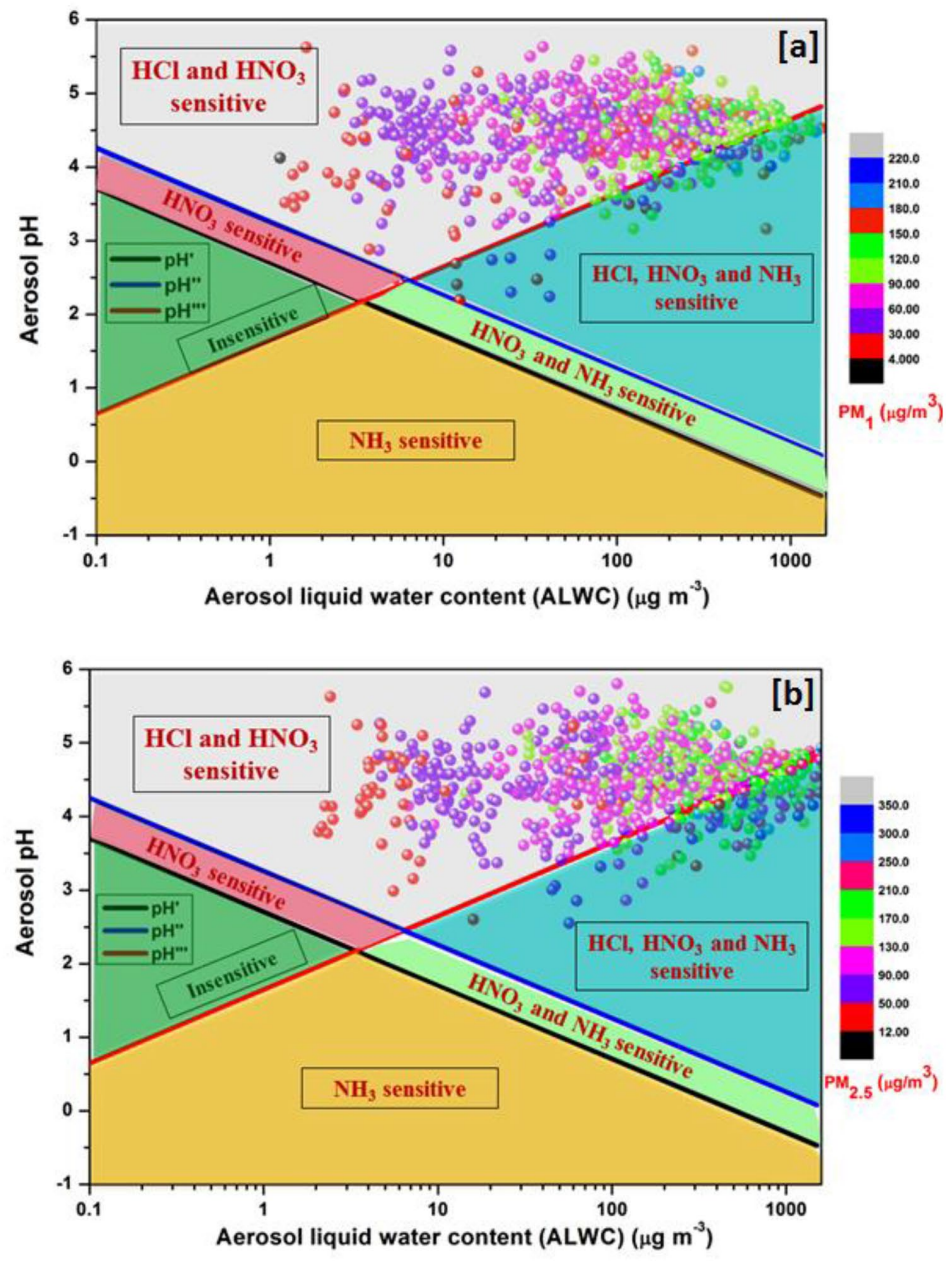
The conceptual thermodynamical framework with the "coordinates" being pH and aerosol liquid water content (ALWC). Aerosols are sensitive to HCl, HNO_3_, and NH_3_ concentrations. The black, blue, and red lines define the "characteristic pH" where chemical regimes are separated by a pre-defined threshold value of ε = 10%. Above pH′, pH″, and pH‴ aerosols are sensitive to the variation in precursors like HCl, HNO_3_, and NH_3_, and below which aerosols are deemed insensitive to variations in precursors. The six chemical regimes of (**a**) PM_1_ and (**b**) PM_2.5_ aerosols are shown in different color shades, with the observational data points colored by mass concentrations (µg m^−3^) of PM_1_ and PM_2.5_ monitored during the Winter Fog Experiment (WiFEx) field campaign of 2017–18.

We explored a thermodynamically consistent mathematical framework to reduce ammonium chloride and ammonium nitrate concentrations in the IGP. Following Eqs. (5–13), and using pH and ALWC as coordinates, we defined six "sensitivity regimes" in Fig. 2, where aerosols are sensitive to HCl, HNO_3_, and NH_3_ perturbations. As chloride dominates the inorganic mass fraction of fine aerosol in the IGP, we specifically investigate the aerosol mass sensitivity to HCl emissions to define an "HCl sensitive regime". This is significantly different from studies conducted in China, USA, and Europe where researchers have investigated aerosol sensitivity to only HNO_3_ and NH_3_ and did not investigate the "HCl sensitive regime"^4–6^.

The "sensitivity regimes" are shown in different colors, where 1100 hourly observational data points are plotted to check the instantaneous response of aerosol loading to HCl, HNO_3_, and NH_3_ variability. Figure 2a,b show PM_1_ and PM_2.5_ aerosols to remain in the  gray shaded region, where aerosols respond proportionally to changes in the HCl and HNO_3_ emissions but tend to be insensitive to NH_3_ emissions. The off-white region is defined as an "HCl and HNO_3_ sensitive regime", indicating that HCl and HNO_3_ reduction would be the most effective pathway in controlling aerosol pollution over IGP. The blue-shaded region is defined as an "HCl, HNO_3_, and NH_3_ sensitive regime", where aerosols are sensitive to HCl, HNO_3_, and NH_3_. It can be seen that very few observational data points fall in this regime, in contrast to the USA, where most of the aerosols fall in this regime^4^. During winter, ammonia concentration is much higher than HCl and HNO_3_ in IGP, but fine aerosols are not sensitive to NH_3_ variations. Instead, HCl and HNO_3_ are by far the limiting factors in aerosol loading, which should be controlled by controlling the major HCl and NO_x_ emissions over IGP.

In Fig. 2a,b, all the data points are color-coded with PM_1_ and PM_2.5_ concentrations, respectively, demonstrating that higher aerosol loading is often associated with higher ALWC. Interestingly, the ALWC usually ranges between tens to hundreds of micrograms per cubic meter in climatic regions like China and USA^4–6^, but over IGP the ALWC is an order of magnitude higher, sometimes reaching ~ 2000 to 2400 µg m^−3^ for PM_1_ and PM_2.5_. This high ALWC significantly influences the loading of SIA and causes a reduction in visibility as evidenced by Henry's law, which shows that particles with high ALWC would take up more gaseous pollutants, and the equilibrium would lead to an increase in their water content and the formation of more secondary aerosols like ammonium chloride and ammonium nitrate^43–46^.

In this work, we explore the physical, chemical, and thermodynamical processes influencing aerosol loading by considering the dominant impact of multiphase and heterogeneous chemical processes on the aerosol growth processes in the wintertime polluted atmosphere in the IGP. From a thermodynamic perspective, it can be seen that acidic gases are first absorbed on the surface layer of aerosols, and heterogeneous reactions rapidly occur at the surface resulting in the rapid increase in secondary aerosol mass concentrations. The newly formed particle mass then gets dispersed through the liquid phase in high ALWC, where multiphase reactions govern. The wintertime ambient atmosphere thermodynamically favors the condensation of species like nitrate and chloride. The vapor pressure of HNO_3_ and HCl decreases with temperature exponentially, which reacts with lofted NH_3_ to stabilize the nitrate and chloride in the particle phase^47^. The higher surface-area-to-volume ratio of PM_1_ than PM_2.5_ suggests the heterogeneous chemistry in PM_1_ to be more crucial than PM_2.5_.

This study significantly differs from studies conducted in India and other parts of the world. For example, recent studies conducted in Europe, the USA, and China suggested that ammonia reduction is more cost-effective than NO_x_ reduction and would be the most effective pathway to reduce aerosol loading^4,38,48^. But in this study, we argue that the sensitivity and effectiveness of the adopted reduction mechanisms are more crucial than the cost-effectiveness. If aerosols are not sensitive to the reductions of a specific precursor, then the cost-effectiveness would not assist in developing an effective mitigation policy.

While studies in India, like Gunthe et al.^24^ have used a fixed concentration of total chloride (i.e., HCl + Cl^−^ = 25 µg m^−3^) for the ISORROPIA-II simulations. This average value does not describe the actual variability of ambient chloride concentrations during the wintertime pollution episodes in Delhi. Instead of this average value, we used measured HCl + Cl^−^ concentrations that varied between 2.11–112.21 µg m^−3^ and 2.31–186.31 µg m^−3^ for PM_1_ and PM_2.5,_ respectively. Thermodynamic model simulations with these high input concentrations helped to capture the different atmospheric concentrations, like clear atmosphere and dense foggy conditions^20^.

In addition, the total nitrate (HNO_3_ + NO_3_^−^) of PM_1_ (2.33–59.96 µg m^−3^) and PM_2.5_ (2.49–85.58 µg m^−3^_)_ also showed considerable variability during the study period. The total ammonia (NH_3_ + NH_4_^+^) varied between 2.15–120.98 and 3.22–159.39 µg m^−3^ for PM_1_ and PM_2.5,_ respectively. Usage of these measured concentrations as the model input can make the thermodynamic model calculations more reliable, capturing the ambient variability more accurately.

To emphasize how sensitive model simulations are to the gas and aerosol phase inputs, the thermodynamic model can reallocate some small amount of NH_4_^+^ into NH_3_ + H^+^, to make aerosol pH, aerosol liquid water content (ALWC) and phase partitioning ratio self-consistent^26^. For example, the transfer of just 1 nmol m^−3^ of NH_4_^+^ into NH_3_ and H^+^ can be enough to reduce the pH by several units. To conclude, the results that we present illustrate the importance of using thermodynamically consistent sensitivity analysis to effectively address the particulate matter pollution mitigation problem in the Indian region.

## Conclusions

This study presents a thermodynamically consistent "roadmap" for effective aerosol mitigation in the Indo-Gangetic Plain (IGP). The proposed framework categorically considers aerosol acidity (pH) and aerosol liquid water content (ALWC) as the governing parameters that modulate the mass loading of aerosols. The mass loading of PM_1_ and PM_2.5_ increases with RH, attributing to the water uptake by hygroscopic constituents and enhanced multiphase reactions in highly humid conditions resulting in visibility reduction.

The identified high ammonium chloride concentration is a special characteristic of air pollution in India, and our results show that aerosols with higher chloride mass fraction absorb more water from the air, triggering the positive feedback between water uptake and enhanced growth of aerosols. This augments the particle mass loading and demonstrates the role of ALWC in the growth of PM_1_ and PM_2.5_ aerosols.

It is crucial to note that the major errors inherent with thermodynamic equilibrium modeling are derived from two assumptions, i.e., internal mixing and gas-aerosol equilibria, and the analytic error in monitoring individual species cannot be evaluated in the absence of specifically designed quality-control experiments.

This study is in significant contrast to any previous studies conducted in India. For instance, due to the lack of observations, studies like Gunthe et al.^24^, and Chen et al.^28^ have used fixed concentrations (25 µg m^−3^) of HCl + Cl^−^ concentrations for the ISORROPIA-II simulations, which could make the thermodynamic model calculations less constrained. As thermodynamic equilibrium significantly depends on both gas and particle phase concentrations, it was much needed to use simultaneously measured gas and aerosol concentrations as model input. So, we used simultaneously measured total chloride (HCl + Cl^−^), total nitrate (HNO_3_ + NO_3_^−^), and total ammonia (NH_3_ + NH_4_^+^) concentrations as model input, which can assist in capturing the ambient variability more accurately. The sigmoidal curves and the sensitivity analysis in Fig. 1a–d show the significance of model-derived parameters like pH and ALWC in modulating the phase-partitioning and particle-phase loading of chloride and nitrate over a location like Delhi.

The sensitivity analyses and statistical parameters like Mean Bias (MB), Normalized Mean Bias (NMB), and Root Mean Square Error (RMSE) presented in this study illustrate the necessity of accounting the actual ambient measurements of gas and aerosol species in the ISORROPIA-II thermodynamic model simulations. For example, the high NMB and RMSE values in pH and ALWC show that the fixed HCl + Cl^−^ = 25 µg m^−3^ cannot represent the real-world variabilities of 2.11–112.21 µg m^−3^ and 2.31–186.31 µg m^−3^ for PM_1_ and PM_2.5_, respectively.

In this study, we investigate the sensitivity of ammonium chloride and ammonium nitrate loading to reductions in gaseous precursors like HCl, HNO_3_, and NH_3_ as a way to reduce PM_1_ and PM_2.5_ mass loading. Six "sensitivity regimes" are defined as (a) "HNO_3_ sensitive", (b) "HCl and HNO_3_ sensitive", (c) "HCl, NH_3_ and HNO_3_ sensitive", (d) "HNO_3_ and NH_3_ sensitive", (e) "NH_3_ sensitive", and (f) "insensitive" to explore the sensitivity of gaseous precursors in the formation of fine aerosols. The application of the framework to the observational dataset shows PM_1_ and PM_2.5_ aerosols to fall in the "HCl and HNO_3_ sensitive regime", emphasizing that HCl and HNO_3_ reductions would be the most effective pathway to reduce aerosol loading over IGP.

The thermodynamic control suggests that although NH_3_ concentrations are much higher than HCl and HNO_3_, the NH_3_ reduction should not be explicitly prioritized to mitigate aerosols. This is in disparity with the existing aerosol mitigation strategies over IGP, which are mostly ineffective and thermodynamically unfavorable^49,50^. Our study differs from similar studies in China, despite the fact that there is a very similar range of phase-partitioning characteristics in winter^51^.

To the best of our knowledge, we are the first to examine the thermodynamic situation via the ε values that show mitigating HCl and HNO_3_ is more effective, not ammonia for IGP in wintertime. We also stress that it is important to determine which precursors the aerosol mass concentrations are sensitive to for determining a cost-effective mitigation policy. If aerosols are not sensitive to the reductions of a specific precursor, then the cost-effectiveness would not assist in developing an effective mitigation policy. Following the framework advanced in this study, India may develop its own and more precise solution to its severe air pollution problem during winter.

## Methods

### Formulation of "sensitivity regimes" of aerosols in the IGP

Factors that strongly influence aerosol properties are aerosol acidity (pH) and aerosol liquid water content (ALWC)^47,51^. The direct monitoring of aerosol pH is highly challenging since an established analytical method of directly determining aerosol pH does not exist^26,52^. Previous studies used several proxy methods like H^+^ ion concentration, ion balance, ammonium-to-sulfate ratio, and cation-to-anion ratio to indirectly infer the fine particle pH^53^. Pye et al.^53^ showed that these methods could not estimate aerosol acidity accurately, and a small bias in estimated pH may cause substantial errors in the chemical and thermodynamical properties of aerosols^21,54^. To accurately estimate pH and ALWC, numerous studies have used thermodynamic models like E-AIM, MARS, and EQUISOL. Past studies have shown that ISORROPIA-II can predict pH and ALWC with great accuracy^54–56^.

The estimated pH and ALWC of PM_1_ and PM_2.5_ are used as coordinates to represent aerosols in different "sensitivity regimes" where aerosol mass is sensitive to HNO_3_, HCl, and/or NH_3_ variability^4^. Three parameters Ψ, Ω, and φ are defined as:

4 $$\Psi =\frac{{H}_{H{NO}_{3}}^{*} R T \times 0.987 \times {10}^{-14} }{{\gamma }_{{H}^{+}}{\gamma }_{{NO}_{3}^{-}}}$$

5 $$\Omega =\frac{{H}_{HCl}^{*} R T \times 0.987 \times {10}^{-14} }{{\gamma }_{{H}^{+}}{\gamma }_{{Cl}^{-}}}$$

6 $$\varphi =\frac{{\gamma }_{{H}^{+}} }{{\gamma }_{{NH}_{4}^{+}}} {H}_{{NH}_{3}}^{*} R T \times 0.987 \times {10}^{-14}$$

Substituting these three parameters in Eqs. (1–3), the partitioning ratio of ε(NO_3_^−^), ε(Cl^−^), and ε(NH_4_^+^) can be expressed in terms of Ψ, Ω, φ, and as a function of ALWC, like

7 $$\varepsilon \left({NO}_{3}^{-}\right)=\frac{\Psi \times {ALWC}_{i}}{\left[{H}^{+}\right]+ \Psi \times {ALWC}_{i}} ,$$

8 $$\varepsilon \left({Cl}^{-}\right)=\frac{\Omega \times {ALWC}_{i}}{\left[{H}^{+}\right]+ \Omega \times {ALWC}_{i}} ,$$

and

9 $$\varepsilon \left({NH}_{4}^{+}\right)=\frac{\varphi \times \left[{H}^{+}\right] \times {ALWC}_{i}}{1+ \varphi \times \left[{H}^{+}\right] \times {ALWC}_{i}}.$$

To check the sensitivity of these partitioning fractions to aerosol pH and ALWC, we assume a characteristic partitioning ratio (ε) value of 0.1 (10%) and define the threshold value of ε(NO_3_^−^), ε(Cl^−^), and ε(NH_4_^+^) as α, β, and γ^4^. Above the threshold value (ε = 10%) the PM_1_ and PM_2.5_ aerosols are sensitive to HNO_3_, HCl, or NH_3_ emissions.

Based on these "characteristic" threshold values, we define "characteristic acidity" for nitrate, (pH'), chloride (pH") and ammonium (pH"'), respectively, as

10 $$p{H}{\prime}=-\mathrm{log}[(\frac{1-\alpha }{ \alpha }) \Psi \times {ALWC}_{i}],$$

11 $$p{H}^{{\prime}{\prime}}=\mathrm{log} [(\frac{1-\beta }{ \beta }) \varphi \times {ALWC}_{i}],$$

and

12 $$p{H}^{{\prime}{\prime}{\prime}}=\mathrm{log} [(\frac{1-\gamma }{ \gamma }) \Omega \times {ALWC}_{i}],$$

which vary logarithmically with ALWC_i_.

Using these characteristic values as coordinates, six "sensitivity regimes" are defined as:

- Regime I: pH < pH′, pH″ and pH > pH‴, where aerosol mass concentration is not sensitive to HNO_3_, HCl or NH_3_ change and defined as "insensitive",
- Regime II: pH > pH′, pH < pH″ and pH > pH‴, where aerosol mass concentration is sensitive to HNO_3_ and not sensitive to HCl and NH_3_ perturbations and defined as "HNO_3_ sensitive",
- Regime III: pH > pH′, pH″ and pH > pH‴, where aerosol mass concentration is sensitive to HCl, HNO_3_ and not sensitive to NH_3_ perturbations and defined as "HCl and HNO_3_ sensitive",
- Regime IV: pH > pH′, pH″ and pH < pH‴, where aerosol mass concentration is sensitive to HCl, HNO_3,_ and NH_3_ perturbations and defined as "HCl, NH_3_ and HNO_3_ sensitive",
- Regime V: pH > pH′ and pH < pH″, pH‴, where aerosol mass concentration is sensitive to HNO_3_ and NH_3_ and not sensitive to HCl and defined as "HNO_3_ and NH_3_ sensitive", and
- Regime VI: pH < pH′, pH″ and pH < pH‴, where aerosol mass concentration is sensitive to NH_3_ and not sensitive to HCl and HNO_3_ and defined as "NH_3_ sensitive".

### Supplementary Information

Supplementary Information.

## Data Availability

The ISORROPIA-II thermodynamic equilibrium model code is available at http://isorropia.epfl.ch. The data used to prepare this manuscript can be found at https://doi.org/10.17605/OSF.IO/6HGS7.
