# Supplementary material for: Thermodynamical framework for effective mitigation of high aerosol loading in the Indo-Gangetic Plain during winter
Source: Sci Rep. 2023 Aug 22;13:13667. doi: 10.1038/s41598-023-40657-w (PMC10444748; doi:10.1038/s41598-023-40657-w)
Supplement: Supplementary file 1 — Supplementary Information. [file 41598_2023_40657_MOESM1_ESM.docx]

**Supporting Online Material**

**Thermodynamical framework for effective mitigation of high aerosol loading in the Indo-Gangetic Plain during winter**

Prodip Acharja^1, 2^, Sachin D. Ghude^1*^, Baerbel Sinha^3*^, Mary Barth^4^, Gaurav Govardhan^1^, Rachana Kulkarni^5^, Vinayak Sinha^3^, Rajesh Kumar^4^, Kaushar Ali^1^, Ismail Gultepe^6,7^, Jean-Eudes Petit^2^, Madhavan Nair Rajeevan^8^

^1^Indian Institute of Tropical Meteorology, Ministry of Earth Sciences, India

^2^Laboratoire des Sciences du Climat et de l’Environnement, LSCE, Gif-sur-Yvette, CNRS, France

^3^Department of Earth and Environmental Sciences, Indian Institute of Science Education and Research Mohali, Punjab, India

^4^National Center for Atmospheric Research, Boulder, CO, 80307, USA

^5^Savitribai Phule Pune University, Pune, India 411007

^6^Ontario Technical University, Engineering and Applied Science, Oshawa, Ontario, Canada

^7^University of Notre Dame, Civil and Environ. Eng and Earth Sciences, Notre Dame, IN, USA

^8^Ministry of Earth Science, Lodhi Road, New Delhi, India

Correspondence to:

*Sachin D. Ghude

Email: [sachinghude@tropmet.res.in](mailto:sachinghude@tropmet.res.in);

*Baerbel Sinha

Email: [bsinha@iisermohali.ac.in](mailto:bsinha@iisermohali.ac.in);

This file includes

Supplementary text

Table S1-S4

Figure S1

Supplementary text

**1. Data and measurements**

**1.1 Measurement site**

The gas and particle-phase species measurements were conducted at the Indira Gandhi International Airport (IGIA), Delhi (28.56° N, 77.09°E, 237 m asl) under the Winter Fog Experiment (WiFEX) field campaign (Ghude et al., 2017). The measurement site fairly represents the general characteristics of highly polluted and densely populated urban locations in the IGP (Sinha et al., 2019; Ali et al., 2019). The measurements were conducted from 19 December 2017 to 10 February 2018 and a detailed description of the observation site and the prominent weather features associated with the winter period in Delhi has been well described in our earlier publications Ali et al., 2019; Acharja et al., 2021.

**1.2 Instrumentation**

**1.2.1 Measurements of trace gases and inorganic constituents of PM_1_, PM_2.5_**

In this study, the measurement of trace gases (HCl, HONO, HNO_3_, SO_2_, and NH_3_) and the inorganic constituents (Cl^-^, NO_3_^-^, SO_4_^2-^, Na^+^, NH_4_^+^, K^+^, Ca^2+^, and Mg^2+^) of fine particulates (PM_1_ and PM_2.5_) were simultaneously conducted at hourly resolution using Monitor for Aerosol and Gases in ambient Air (MARGA-2S, Make: Metrohm Applikon B.V, Netherlands) instrument (Rumsey et al., 2014).

The instrument is an assembly of equipment consisting of sampling unit, denuder for separating trace gases from the particles, aerosol collector, and analytical unit (Makkonen et al., 2012). MARGA-2S can provide a physicochemical characterization of particle matter along with trace gases on hourly time resolution (Schaap et al., 2011). In the measurement system, ambient air is first drawn through the PM_1_, PM_2.5_ inlets that pass through horizontal wet rotating denuder (WRD), and gases get diffused into an aqueous film. The aerosol gets collected in the steam jet aerosol collector (SJAC), where water-soluble aerosols grow in a supersaturated environment (Stieger et al., 2018). The water-soluble inorganic ions are quantified with two ion chromatography systems (anion + cation) using the principle presented by Wyers et al., 1993. The MARGA-2S instrument was installed on the first floor of the building, whereas PM_1_ and PM_2.5_ impactors were installed on the terrace of the building nearly 8m above the ground and 2m above the roof surface. The instrument was continuously calibrated during its operation to ensure the quality of observation. A detailed description of the MARGA-2S instrument, its operating principle, calibration method, and study location is discussed in our previous publication (Acharja et al., 2020). Meteorological parameters like temperature, relative humidity, wind speed, and wind direction were monitored with co-located automatic weather station (AWS).

**2. Methodology**

**2.1 Thermodynamic equilibrium model ISORROPIA-II**

The thermodynamic state of aerosols depends on ionic strength, dissociation of electrolyte, temperature of the solution, pH of aerosol, water activity, surface tension, density, and hygroscopicity (Kim et al., 1993; Tilgner et al., 2021). In the present study, ISORROPIA-II (Cl^−^ - NO_3_^−^ - SO_4_^2−^ - NH_4_^+^ - Na^+^ - K^+^ - Mg^2+^ - Ca^2+^ - H_2_O system) model was used (Fountoukis and Nenes, 2007; **Weber et al., 2016;** Liu et al., 2017). In the model, the equilibrium is characterized by simultaneously considering a set of nonlinear algebraic equations coupled with appropriate electro-neutrality and mass conservation equations using the Newton-Raphson and bisectional methods (Ansari and Pandis., 1999). It is also assumed that the system is closed with respect to the total constituents, and the Gibbs free energy is at the global minimum. At this state, the variation in entropy concerning neighboring states is zero, and the system may remain at this energy level for arbitrarily long times.

The observational dataset of concentration of total ammonium (TNH_4_=NH_3_ + NH_4_^+^), total nitrate (TNO_3_=HNO_3_ + NO_3_^-^), and total chloride (TCl=HCl + Cl^-^) along with Na^+^, K^+^, Ca^2+^, and Mg^2+^ were used as inputs to run the model. The model can run in the "backward" mode based on the particle-phase data and in the "forward" mode based on the total (particle + gas) phase concentration data (Xu et al., 2015; **Yao et al., 2007; Guo et al., 2017; Song et al., 2007**). Studies have shown the "forward" mode to be more accurate and less sensitive to the measurement uncertainties compared to the "backward" mode (Hennigan et al., 2015; Bian et al., 2014). In the "forward" mode, the aerosols are assumed to exist in a metastable or aqueous state. These aqueous particles being larger than dry crystalline solids, generally scatter more light, degrade visibility, and influence mass transfer kinetics of trace gases (Rood et al., 1989).

The particle acidity (pH) is estimated using the following equation:

$$pH={-log}_{10}\frac{{1000\times[H}_{air}^{+}]}{ALWC} \left( S1 \right)$$

where [H_air_^+^] concentration and aerosol liquid water content (ALWC) are the ISORROPIA-II model outputs (Liu et al., 2014). In the model, the ALWC was estimated by considering the Raoult effect, curvature effect, and water uptake property (Bian et al., 2014; Hennigan et al., 2015). The water uptake of aerosols is calculated with the Zdanovskii–Stokes–Robinson (ZSR) hypothesis (Stokes and Robinson, 1966)

$$A=\sum_{i} \frac{M_{i}}{m_{oi}{(a}_{w})} (S2)$$

where M_i_ is the mole concentration of the electrolyte i (mol m^−3^), m_oi_(a_w_) is the corresponding molality of the binary solution of the electrolyte i under the same a_w_ (Seinfeld and Pandis, 2008). At the phase equilibrium a_w_ is equal to the ambient RH:

$$a_{w}=RH (S3)$$

This approach ignores the contribution of organic species (e.g., oxalate, formate, acetate) to ALWC, but studies have shown that organics do not significantly affect the aerosol liquid water content (Ziemba et al., 2007; Wang et al., 2019).

In this study, the data of RH in the 30–95% range were considered for the model simulation and data of RH > 95% were excluded since the exponential growth in ALWC with RH introduces large pH uncertainties (Guo et al., 2015). During the study period, the ambient humidity was high (≥50%), much higher than the efflorescence RH (10-30 %), so the "forward" mode consideration and the assumption that aerosols were in a completely deliquesced phase and remained at the "metastable" side of the hysteresis is reasonable. The model-derived pH of PM_2.5_ was compared to make a comprehensive analysis of the model simulation.

**2.2 Gas-particle partitioning ratio**

The gas-to-particle partitioning and formation of secondary aerosols are significantly impacted by pH, ALWC, and T. High ALWC results in uptake of a gaseous species onto liquid and phase partitioning depending on the multiphase and heterogeneous reaction rates and the measurement or module of the model used (Nemitz et al., 2004; Hanson et al., 1994; **Murphy et al., 2017**). The partitioning ratio of ammonium [ε(NH_4_^+^)], nitrate [ε(NO_3_^-^)], and chloride [ε(Cl^-^)] can be estimated using the following equations:

$$\varepsilon\left( {NH}_{4}^{+} \right)=\frac{\left[ {NH}_{4}^{+} \right]}{\left[ {NH}_{3} \right]+\left[ {NH}_{4}^{+} \right]} (S4)$$

$$\varepsilon\left( {NO}_{3}^{-} \right)=\frac{\left[ {NO}_{3}^{-} \right]}{\left[ {HNO}_{3} \right]+\left[ {NO}_{3}^{-} \right]} (S5)$$

$$\varepsilon\left( {Cl}^{-} \right)=\frac{\left[ {Cl}^{-} \right]}{\left[ HCl \right]+\left[ {Cl}^{-} \right]} (S6)$$

where [NH_4_^+^], [NO_3_^-^], [Cl^-^], [NH_3_], [HNO_3_], [HCl] are the molar concentrations (μmol m^−3^) of particulate and gaseous species respectively.

**2.3 Uncertainty analysis**

To thoroughly investigate and reduce the uncertainty in the gas and aerosol species measurements, we have carefully monitored the major parameters like shelter temperature, accuracy of the internal standard, liquid sample volume, and chromatogram peak shapes during the study. For example, the MARGA-2S instrument was installed in a temperature controlled (25 ± 5 °C) shelter, and the lithium bromide (LiBr) internal standard was monitored for every sample following the manufacturer's specifications (Rumsey et al., 2014; Allen et al., 2015). The data analysis also considers the limit of detection (LOD) of measured species (Table S3). As most of the major species concentrations during winter were much higher than the LOD, the uncertainty due to LOD is significantly less in our study.

In addition to these, we have also calculated uncertainty by considering the 95% confidence interval (CI) and its upper and lower limits, which is traditionally the most used confidence interval in the literature. This relates to the generally accepted level of statistical significance of 5% or p < 0.05. The ISORROPIA-II thermodynamical model was then run with these three different values for each variable (95% CI and its upper and lower limit), and the model-derived ALWC and pH were statistically evaluated in Table S4.

The agreement between the predicted and measured partitioning ratios has been evaluated by estimating the mean bias (MB) and normalized mean bias (NMB), root mean square error (RMSE) using the following equations:

$$MB=\frac{1}{N}\times\sum_{i=1}^{N} \left( M_{i}-O_{i} \right) (S7)$$

$$NMB=\frac{\sum_{i=1}^{N} \left( M_{i}-O_{i} \right)}{\sum_{i=1}^{N} O_{i}}\times100\% (S8)$$

$$RMSE=\sqrt{\frac{1}{n} \sum_{1}^{n} {(M_{i}- O_{i})}^{2}} (S9)$$

where, M_i_ and O_i_ are the model predicted and observed data, respectively, and N is the number of data points (Sudheer et al., 2015). These metrics are commonly used to evaluate the model predictions, and negative values for MB and NMB imply lower prediction, whereas positive values show the over-prediction.

Table S1. Studies investigating the atmospheric abundances of inorganics and organic constituents of aerosols from different parts of the world. These studies have shown the contribution of inorganics constituents to be higher than organics during high pollution episodes, unless noted specifically.

| Study Location | Study Period (month/year) | Reference |
| --- | --- | --- |
| Beijing, Shanghai, Guangzhou and Xi'an, China | January-February 2013 | Huang et al., 2014 |
| Beijing, China | 21 Nov 2011 - 20 Jan 2012 | Sun et al., 2013 |
| Longyou, China | 01 January - 5 February 2018 | Bao et al., 2019 |
| Beijing, China | 2014 – 2017 | Li et al., 2019 |
| Sanya, China | 12 April – 14 May, 2017 | Tian et al., 2020 |
| Cabauw, The Netherlands | May 2008 and March 2009 | Mensah et al., 2012 |
| Paris, France | June 2011 – May 2013 | Petit et al., 2015 |
| Kathmandu, Nepal | 5^th^ - 14^th^ January 2018 | Werden et al., 2022 |
| Kanpur, India | January 2007 – March 2008 | Ram et al., 2010 |
| Delhi, India | November 2009 – March 2010 | Perrino et al., 2011 |
| Delhi, India | December 2013 - January 2014 | Pant et al., 2015 |
| Delhi, Varanasi, and Kolkata, India | January – December 2011 | Sharma et al., 2016 |
| Hisar, Delhi, India | January – December 2018 | Bhowmik et al., 2021 |
| Delhi, India | January 2018 | Gani et al., 2019 |

**Table S2.** The measured total (gas + aerosol) concentration of chloride (TCl), nitrate (TNO_3_), and ammonia (TNH_4_), estimated pH, ALWC, and gas-to-particle partitioning ratio (ε) of ε (Cl^-^), ε (NO_3_^-^), and ε (NH_4_^+^) of PM_1_ and PM_2.5_ during the WiFEX campaign period of 2017-18.

| Variables | PM_1_ | PM_2.5_ |
| --- | --- | --- |
| TNO_3_ (µg m^-3^) | 19.74 ± 9.81 | 31.67 ± 14.57 |
| TCl (µg m^-3^) | 22.28 ± 20.57 | 39.71 ± 35.41 |
| TNH_4_ (µg m^-3^) | 43.41 ± 19.81 | 58.04 ± 24.06 |
| Predicted from ISORROPIA-II | | |
| pH | 4.5 ± 0.5 | 4.6 ± 0.5 |
| ALWC (µg m^-3^) | 169 ± 205 | 324 ± 396 |
| Gas-to-particle partitioning ratio (ε) | | |
| ε (Cl^-^ ) | 93 ± 09 % | 96 ± 07 % |
| ε(NO_3_-) | 83 ± 11 % | 89 ± 08 % |
| ε (NH_4_^+^) | 42 ± 17 % | 55 ± 15 % |

**Table S3.** Limits of detection (LOD) of MARGA system for inorganic ionic species and trace gases and gas separation efficiency of the denuder system.

| Sr. No. | Inorganic ions and trace gases | Limit of detection (LOD) | Gas separation efficiency of the denuder system | Reference |
| --- | --- | --- | --- | --- |
| 1 | Cl^-^ | 0.05 µg/m^3^ | - | Allen et al., 2015  Rumsey et al., 2014 |
| 2 | NO_3_^-^ | 0.05 µg/m^3^ | - |  |
| 3 | SO_4_^2-^ | 0.08 µg/m^3^ | - |  |
| 4 | Na^+^ | 0.08 µg/m^3^ | - |  |
| 5 | NH_4_^+^ | 0.08 µg/m^3^ | - |  |
| 6 | K^+^ | 0.10 µg/m^3^ | - |  |
| 7 | Mg^2+^ | 0.10 µg/m^3^ | - |  |
| 8 | Ca^2+^ | 0.08 µg/m^3^ | - |  |
| 9 | HCl | 0.05 µg/m^3^ | 99.7% | Stieger et al., 2019;  Winiwarter et al., 1989;  Possanzini et al., 1983; Wyers et al., 1993;  Berg et al., 2010 |
| 10 | HNO_2_ | 0.08 µg/m^3^ | 99.8% |  |
| 11 | HNO_3_ | 0.05 µg/m^3^ | 99.6% |  |
| 12 | SO_2_ | 0.10 µg/m^3^ | 99.5% |  |
| 13 | NH_3_ | 0.08 µg/m^3^ | 99.3% |  |

**Table S4.** Sensitivity of the ISORROPIA-II model outputs like aerosol acidity (pH), aerosol liquid water content (ALWC) to input concentrations of gas and particle-phase constituents. The statistical evaluations are done following the equations S7 – S9. The evaluated Mean Bias (MB), Normalized Mean Bias (NMB), and Root Mean Square Error (RMSE) for both PM_1_ and PM_2.5_ are mentioned here. We have also conducted statistical evaluation due to the 95% confidence interval upper and lower limit of the major input parameters for both PM_1_ and PM_2.5_.

| Input Parameters | Statistical Evaluation | Aerosol liquid water content (ALWC) | Aerosol acidity (pH) |
| --- | --- | --- | --- |
| PM_1_ | | | |
| HCl + Cl^-^  (25 µg m^-3^) | MB | -13.69 µg m^-3^ | 0.14 |
|  | NMB (%) | -8.34% | 3.18% |
|  | RMSE | 149.69 µg m^-3^ | 0.42 |
| HNO_3_ + NO_3_^-^  (25 µg m^-3^) | MB | -12.97 µg m^-3^ | 0.04 |
|  | NMB (%) | -7.9% | 1.1% |
|  | RMSE | 46.8 µg m^-3^ | 0.83 |
| NH_3_ + NH_4_^+^  (25 µg m^-3^) | MB | -28.29 µg m^-3^ | -0.68 |
|  | NMB (%) | -17.23% | -5.53% |
|  | RMSE | 93.70 µg m^-3^ | 1.22 |
| PM_2.5_ | | | |
| HCl + Cl^-^  (25 µg m^-3^) | MB | -85.83 µg m^-3^ | 0.18 |
|  | NMB (%) | -26.61% | 4.13% |
|  | RMSE | 264.98 µg m^-3^ | 0.55 |
| HNO_3_ + NO_3_-  (25 µg m^-3^) | MB | -19.29 µg m^-3^ | 0.08 |
|  | NMB (%) | -5.98% | 1.76% |
|  | RMSE | 62.49 µg m^-3^ | 0.36 |
| NH_3_ + NH_4_^+^  (25 µg m^-3^) | MB | -140.06 µg m^-3^ | -1.62 |
|  | NMB (%) | -43.4% | -0.36% |
|  | RMSE | 307.81 µg m^-3^ | 2.08 |
| PM_1_ | | | |
| "Forward" mode model inputs with 95% Confidence Intervals upper and lower limits values | MB | -5.62 - 7.04 µg m^-3^ | -0.025 – 0.027 |
|  | NMB (%) | -7.21 - 9.04% | 0.53 - 0.57% |
|  | RMSE | 5.62 - 7.04 µg m^-3^ | 0.025 - 0.027 |
| PM_2.5_ | | | |
| "Forward" mode model inputs with 95% Confidence Intervals upper and lower limits values | MB | -11.6 – 12.4 µg m^-3^ | -0.033 – 0.035 |
|  | NMB (%) | -8.3 – 8.9 % | 0.74 – 0.75% |
|  | RMSE | 11.6 – 12.4 µg m^-3^ | 0.033 – 0.035 |

**Figure S1:**

**
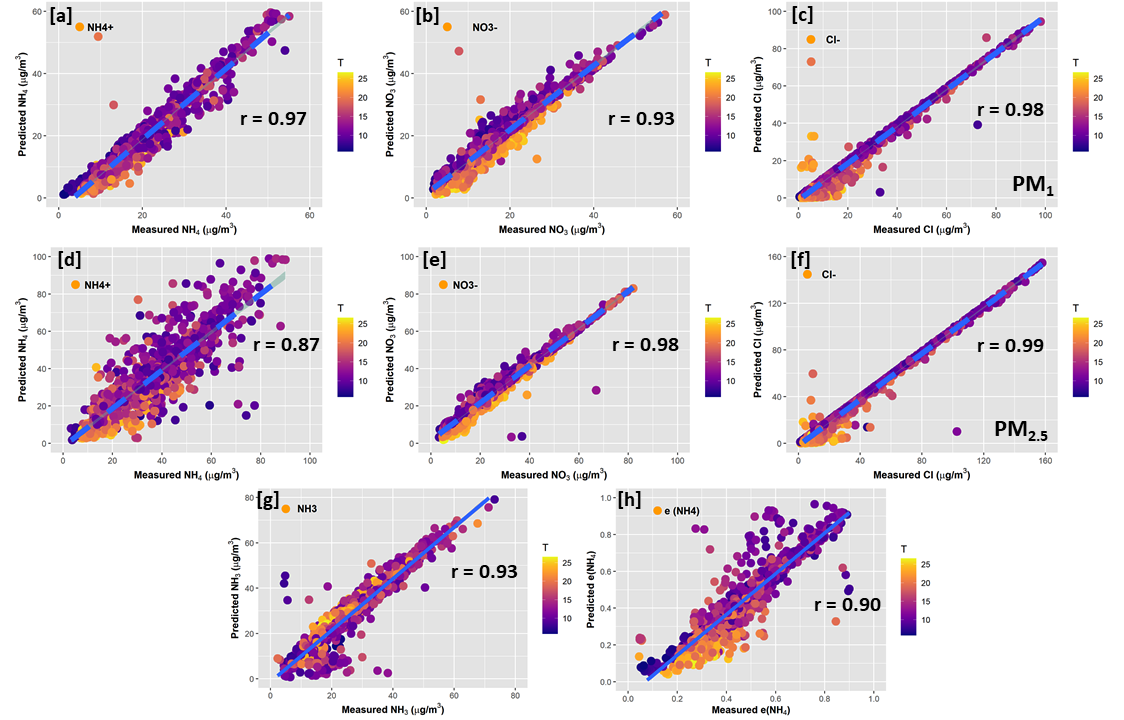
**

**Figure S1:** Comparison of ISORROPIA-II predicted and MARGA-2S measured NH_4_^+^, NO_3_^-^, and Cl^-^ of PM_1_ (a, b, c) and PM_2.5_ (d, e, f), gas-phase NH_3_ (g) and (h) gas-to-particle partitioning ratio [ε(NH_4_^+^)]_._ The correlation (r) between the predicted and measured species concentrations are reported on each panel. Data are color-coded by ambient air temperatures (T).

**References:**

**Allen, H.M., Draper, D.C., Ayres, B.R., Ault, A., Bondy, A., Takahama, S., Modini, R.L., Baumann, K., Edgerton, E., Knote, C. and Laskin, A., 2015. Influence of crustal dust and sea spray supermicron particle concentrations and acidity on inorganic NO_3_^−^ aerosol during the 2013 Southern Oxidant and Aerosol Study. Atmospheric Chemistry and Physics, 15(18), pp.10669-10685.**

**Ansari, A.S. and Pandis, S.N., 2000. The effect of metastable equilibrium states on the partitioning of nitrate between the gas and aerosol phases. Atmospheric Environment, 34(1), pp.157-168.**

**Bao, Z., Chen, L., Li, K., Han, L., Wu, X., Gao, X., Azzi, M. and Cen, K., 2019. Meteorological and chemical impacts on PM2. 5 during a haze episode in a heavily polluted basin city of eastern China. Environmental Pollution, 250, pp.520-529.**

**Berg, J.M., James, D.L., Berg, C.F., Toda, K. and Dasgupta, P.K., 2010. Gas collection efficiency of annular denuders: A spreadsheet-based calculator. Analyticachimicaacta, 664(1), pp.56-61.**

**Bhowmik, H.S., Naresh, S., Bhattu, D., Rastogi, N., Prévôt, A.S. and Tripathi, S.N., 2021. Temporal and spatial variability of carbonaceous species (EC; OC; WSOC and SOA) in PM2. 5 aerosol over five sites of Indo-Gangetic Plain. Atmospheric Pollution Research, 12(1), pp.375-390.**

Bian, Y.X., Zhao, C.S., Ma, N., Chen, J. and Xu, W.Y., 2014. A study of aerosol liquid water content based on hygroscopicity measurements at high relative humidity in the North China Plain. Atmospheric Chemistry and Physics, 14(12), pp.6417-6426.

Gani, S., Bhandari, S., Seraj, S., Wang, D.S., Patel, K., Soni, P., Arub, Z., Habib, G., Hildebrandt Ruiz, L. and Apte, J.S., 2019. Submicron aerosol composition in the world's most polluted megacity: the Delhi Aerosol Supersite study. Atmospheric Chemistry and Physics, 19(10), pp.6843-6859.

Guo, H., Xu, L., Bougiatioti, A., Cerully, K.M., Capps, S.L., Hite Jr, J.R., Carlton, A.G., Lee, S.H., Bergin, M.H., Ng, N.L. and Nenes, A., 2015. Fine-particle water and pH in the southeastern United States. Atmospheric Chemistry and Physics, 15(9), pp.5211-5228.

Guo, H., Otjes, R., Schlag, P., Kiendler-Scharr, A., Nenes, A. and Weber, R.J., 2017. Effectiveness of ammonia reduction on control of fine particle nitrate. Atmospheric Chemistry and Physics, 18(16), pp.12241-12256.

Hanson, D.R., Ravishankara, A.R. and Solomon, S., 1994. Heterogeneous reactions in sulfuric acid aerosols: A framework for model calculations. Journal of Geophysical Research: Atmospheres, 99(D2), pp.3615-3629.

Hennigan, C.J., Izumi, J., Sullivan, A.P., Weber, R.J. and Nenes, A., 2015. A critical evaluation of proxy methods used to estimate the acidity of atmospheric particles. Atmospheric Chemistry and Physics, 15(5), pp.2775-2790.

Huang, R.J., Zhang, Y., Bozzetti, C., Ho, K.F., Cao, J.J., Han, Y., Daellenbach, K.R., Slowik, J.G., Platt, S.M., Canonaco, F. and Zotter, P., 2014. High secondary aerosol contribution to particulate pollution during haze events in China. Nature, 514(7521), pp.218-222.

**Kim, Y.P., Seinfeld, J.H. and Saxena, P., 1993. Atmospheric gas-aerosol equilibrium I. Thermodynamic model. Aerosol Science and Technology, 19(2), pp.157-181.**

**Li, H., Cheng, J., Zhang, Q., Zheng, B., Zhang, Y., Zheng, G. and He, K., 2019. Rapid transition in winter aerosol composition in Beijing from 2014 to 2017: response to clean air actions. Atmospheric Chemistry and Physics, 19(17), pp.11485-11499.**

**Makkonen, U., Virkkula, A., Mäntykenttä, J., Hakola, H., Keronen, P., Vakkari, V. and Aalto, P.P., 2012. Semi-continuous gas and inorganic aerosol measurements at a Finnish urban site: comparisons with filters, nitrogen in aerosol and gas phases, and aerosol acidity. Atmospheric Chemistry and Physics, 12(12), pp.5617-5631.**

**Mensah, A.A., Holzinger, R., Otjes, R., Trimborn, A., Mentel, T.F., Ten Brink, H., Henzing, B. and Kiendler-Scharr, A., 2012. Aerosol chemical composition at Cabauw, The Netherlands as observed in two intensive periods in May 2008 and March 2009. Atmospheric chemistry and physics, 12(10), pp.4723-4742.**

**Murphy, J.G., Gregoire, P.K., Tevlin, A.G., Wentworth, G.R., Ellis, R.A., Markovic, M.Z. and VandenBoer, T.C., 2017. Observational constraints on particle acidity using measurements and modelling of particles and gases. Faraday discussions, 200, pp.379-395.**

**Nemitz, E., Sutton, M.A., Wyers, G.P. and Jongejan, P.A.C., 2004. Gas-particle interactions above a Dutch heathland: I. Surface exchange fluxes of NH 3, SO 2, HNO 3 and HCl. Atmospheric Chemistry and Physics, 4(4), pp.989-1005.**

**Pant, P., Shukla, A., Kohl, S.D., Chow, J.C., Watson, J.G. and Harrison, R.M., 2015. Characterization of ambient PM2. 5 at a pollution hotspot in New Delhi, India and inference of sources. Atmospheric environment, 109, pp.178-189.**

**Perrino, C., Tiwari, S., Catrambone, M., Dalla Torre, S., Rantica, E. and Canepari, S., 2011. Chemical characterization of atmospheric PM in Delhi, India, during different periods of the year including Diwali festival. Atmospheric Pollution Research, 2(4), pp.418-427.**

**Petit, J.E., Favez, O., Sciare, J., Crenn, V., Sarda-Esteve, R., Bonnaire, N., Močnik, G., Dupont, J.C., Haeffelin, M. and Leoz-Garziandia, E., 2015. Two years of near real-time chemical composition of submicron aerosols in the region of Paris using an Aerosol Chemical Speciation Monitor (ACSM) and a multi-wavelength Aethalometer. Atmospheric Chemistry and Physics, 15(6), pp.2985-3005.**

**Possanzini, M., Febo, A. and Liberti, A., 1983.New design of a high-performance denuder for the sampling of atmospheric pollutants. Atmospheric Environment (1967), 17(12), pp.2605-2610.**

**Ram, K., Sarin, M.M. and Tripathi, S.N., 2010. A 1 year record of carbonaceous aerosols from an urban site in the Indo‐Gangetic Plain: Characterization, sources, and temporal variability. Journal of Geophysical Research: Atmospheres, 115(D24).**

**Rood, M.J., Shaw, M.A., Larson, T.V. and Covert, D.S., 1989. Ubiquitous nature of ambient metastable aerosol. Nature, 337(6207), pp.537-539.**

**Rumsey, I.C., Cowen, K.A., Walker, J.T., Kelly, T.J., Hanft, E.A., Mishoe, K., Rogers, C., Proost, R., Beachley, G.M., Lear, G. and Frelink, T., 2014. An assessment of the performance of the Monitor for AeRosols and GAses in ambient air (MARGA): a semi-continuous method for soluble compounds. Atmospheric Chemistry and Physics, 14(11), pp.5639-5658.**

**Schaap, M., Otjes, R.P. and Weijers, E.P., 2011. Illustrating the benefit of using hourly monitoring data on secondary inorganic aerosol and its precursors for model evaluation. Atmospheric Chemistry and Physics, 11(21), pp.11041-11053.**

**Sharma, S.K., Mandal, T.K., Srivastava, M.K., Chatterjee, A., Jain, S., Saxena, M., Singh, B.P., Sharma, A., Adak, A. and K. Ghosh, S., 2016. Spatio-temporal variation in chemical characteristics of PM 10 over Indo Gangetic Plain of India. Environmental Science and Pollution Research, 23, pp.18809-18822.**

**Song, S., Gao, M., Xu, W., Shao, J., Shi, G., Wang, S., Wang, Y., Sun, Y. and McElroy, M.B., 2018. Fine-particle pH for Beijing winter haze as inferred from different thermodynamic equilibrium models. Atmospheric Chemistry and Physics, 18(10), pp.7423-7438.**

**Stieger, B., Spindler, G., Fahlbusch, B., Müller, K., Grüner, A., Poulain, L., Thöni, L., Seitler, E., Wallasch, M. and Herrmann, H., 2018. Measurements of PM10 ions and trace gases with the online system MARGA at the research station Melpitz in Germany–A five-year study. Journal of Atmospheric Chemistry, 75(1), pp.33-70.**

**Sudheer, A.K. and Rengarajan, R., 2015. Time-resolved inorganic chemical composition of fine aerosol and associated precursor gases over an urban environment in western India: gas-aerosol equilibrium characteristics. Atmospheric Environment, 109, pp.217-227.**

**Sun, Y., Wang, Z., Fu, P., Jiang, Q., Yang, T., Li, J. and Ge, X., 2013. The impact of relative humidity on aerosol composition and evolution processes during wintertime in Beijing, China. Atmospheric Environment, 77, pp.927-934.**

**Tian, J., Wang, Q., Han, Y., Ye, J., Wang, P., Pongpiachan, S., Ni, H., Zhou, Y., Wang, M., Zhao, Y. and Cao, J., 2020. Contributions of aerosol composition and sources to particulate optical properties in a southern coastal city of China. Atmospheric Research, 235, p.104744.**

**Tilgner, A., Schaefer, T., Alexander, B., Barth, M., Collett Jr, J.L., Fahey, K.M., Nenes, A., Pye, H.O., Herrmann, H. and McNeill, V.F., 2021. Acidity and the multiphase chemistry of atmospheric aqueous particles and clouds. Atmospheric Chemistry and Physics Discussions, pp.1-82.**

**Werden, B.S., Giordano, M.R., Mahata, K., Islam, M.R., Goetz, J.D., Puppala, S.P., Saikawa, E., Panday, A.K., Yokelson, R.J., Stone, E.A. and DeCarlo, P.F., 2022. Submicron Aerosol Composition and Source Contribution across the Kathmandu Valley, Nepal, in Winter. ACS earth and space chemistry.**

**Winiwarter, W., 1989.A calculation procedure for the determination of the collection efficiency in annular denuders. Atmospheric Environment (1967), 23(9), pp.1997-2002.**

**Wyers, G.P., Otjes, R.P. and Slanina, J., 1993. A continuous-flow denuder for the measurement of ambient concentrations and surface-exchange fluxes of ammonia. Atmospheric Environment. Part A. General Topics, 27(13), pp.2085-2090.**

**Xu, L., Guo, H., Boyd, C.M., Klein, M., Bougiatioti, A., Cerully, K.M., Hite, J.R., Isaacman-VanWertz, G., Kreisberg, N.M., Knote, C. and Olson, K., 2015. Effects of anthropogenic emissions on aerosol formation from isoprene and monoterpenes in the southeastern United States. Proceedings of the National Academy of Sciences, 112(1), pp.37-42.**

**Yao, X., Ling, T.Y., Fang, M. and Chan, C.K., 2007. Size dependence of in situ pH in submicron atmospheric particles in Hong Kong. Atmospheric Environment, 41(2), pp.382-393.**

**Ziemba, L.D., Fischer, E., Griffin, R.J. and Talbot, R.W., 2007. Aerosol acidity in rural New England: Temporal trends and source region analysis. Journal of Geophysical Research: Atmospheres, 112(D10).**
